# Heterogeneous nuclear ribonucleoprotein K is associated with poor prognosis and regulates proliferation and apoptosis in bladder cancer

**DOI:** 10.1111/jcmm.12999

**Published:** 2016-11-10

**Authors:** Xu Chen, Peng Gu, Ruihui Xie, Jinli Han, Hao Liu, Bo Wang, Weibin Xie, Weijie Xie, Guangzheng Zhong, Changhao Chen, Shujie Xie, Ning Jiang, Tianxin Lin, Jian Huang

**Affiliations:** ^1^ Department of UrologySun Yat‐sen Memorial HospitalSun Yat‐sen UniversityGuangzhouChina; ^2^ Guangdong Provincial Key Laboratory of Malignant Tumor Epigenetics and Gene RegulationSun Yat‐Sen Memorial HospitalSun Yat‐Sen UniversityGuangzhouChina

**Keywords:** hnRNPK, bladder cancer, proliferation, apoptosis, transcriptional regulation

## Abstract

Heterogeneous nuclear ribonucleoprotein K (hnRNPK) is an essential RNA‐ and DNA‐binding protein that regulates diverse biological events, especially DNA transcription. hnRNPK overexpression is related to tumorigenesis in several cancers. However, both the expression patterns and biological mechanisms of hnRNPK in bladder cancer are unclear. We investigated hnRNPK expression by immunohistochemistry in 188 patients with bladder cancer, and found that hnRNPK expression levels were significantly increased in bladder cancer tissues and that high‐hnRNPK expression was closely correlated with poor prognosis. Loss‐ and gain‐of‐function assays demonstrated that hnRNPK promoted proliferation, anti‐apoptosis, and chemoresistance in bladder cancer cells *in vitro*, and hnRNPK knockdown suppressed tumorigenicity *in vivo*. Mechanistically, hnRNPK regulated various functions in bladder cancer by directly mediating cyclin D1, G0/G1 switch 2 (*G0S2*), XIAP‐associated factor 1, and ERCC excision repair 4, endonuclease catalytic subunit (*ERCC4*) transcription. In conclusion, we discovered that hnRNPK plays an important role in bladder cancer, suggesting that it is a potential prognostic marker and a promising target for treating bladder cancer.

## Introduction

Bladder cancer is one of the most common cancers and accounts for approximately 429,800 newly diagnosed cases and 165,100 deaths per year worldwide 1. Emerging evidence shows that aberrant cell cycle 2, 3, excessive anti‐apoptosis 3, 4, 5, and chemoresistance 6, 7, 8 signalling are involved in the carcinogenesis and progression of bladder cancer. Previous studies have found that the mechanism of bladder cancer is complex and coregulated by several molecular networks 3, 9, but many of the key elements are not fully understood.

Heterogeneous nuclear ribonucleoprotein K (hnRNPK), a member of the hnRNP family, is an essential RNA‐ and DNA‐binding protein. Structurally, it contains three consecutive K homologue domains that are responsible for RNA or single‐stranded DNA binding, a nuclear localization signal that induces its transport from the cytoplasm to the nucleus, and a nuclear shuttling domain that regulates its translocation to the cytoplasm 10, 11, 12. Biologically, hnRNPK interacts with diverse molecules involved in gene expression and signal transduction, including chromosome remodelling, DNA transcription, RNA processing, RNA splicing, and RNA stability and translation 7, 13.

hnRNPK is overexpressed in human cancers, including colorectal, pancreatic, liver, prostate and renal cancer 14, 15, 16, 17, 18. Heterogeneous nuclear ribonucleoprotein K is thought to play an important role in cancer progression, as high levels of expression correlate with poor clinical outcome 16, 17, 18. Several studies have found that hnRNPK promoted metastases in tumours by up‐regulating matrix metalloproteinase 18, 19, 20. Knockdown of hnRNPK suppressed proliferation in pancreatic and renal cancer 16, 18. In addition, hnRNPK serves as a transcriptional cofactor for the p53 pathway during the DNA damage response 21. However, a recent report showed that, in liver cancer, hnRNPK suppressed apoptosis independent of p53 status by promoting X‐linked inhibitor of apoptosis protein (XIAP) 22. Until now, there has been no report on hnRNPK behaviour in bladder cancer.

In this study, we investigated hnRNPK expression in bladder cancer tissues and analysed its correlation with the clinicopathological characteristics and overall survival of bladder cancer. We also studied the function and mechanism of hnRNPK in bladder cancer cells. Our findings strongly suggest that hnRNPK participates in bladder cancer carcinogenesis and is a potential diagnostic and prognostic marker and a promising therapeutic target.

## Material and methods

### Tissue samples

Four tissue microarrays containing 159 bladder cancer specimens and 92 normal tissues were purchased from Shanghai Outdo Biotech (Shanghai, China) and US Biomax. In 59 cases, the tissue microarray contained the patients' follow‐up data, but the cause of death was unclear. A further 29 bladder cancer specimens, which included the patients' follow‐up data, and 10 normal tissues were obtained from patients undergoing radical cystectomy at Sun Yat‐sen Memorial Hospital between June 2012 and June 2014. All samples were evaluated and histologically diagnosed by expert pathologists. All samples were collected with informed consent according to the Sun Yat‐Sen University internal review and ethics boards. Table S1 lists the patient and tumour demographics.

### Immunohistochemical (IHC) staining and scoring analyses

This experiment was conducted as previously described 23, 24. Briefly, paraffin sections of bladder cancer tissues and normal tissues were first deparaffinized and hydrated. Microwave antigen retrieval was performed for all antibodies, and endogenous peroxidase activity was blocked by incubating the slides in 0.3% H_2_O_2_. After serial incubation with primary antibodies and secondary antibody, sections were developed with peroxidase and 3,3′‐diaminobenzidine tetrahydrochloride. The sections were then counterstained with haematoxylin and mounted in non‐aqueous mounting medium. Anti‐hnRNPK antibody (1:50; sc‐28380; Santa Cruz Biotechnology, Santa Cruz, CA, USA) was used to detect hnRNPK expression in the specimens. Anti‐hnRNPK and anti‐Ki67 antibodies (1:500; Zhongshan Bio‐Tech, Beijing, China) were used to detect hnRNPK and Ki67 expression in mouse tumours. Human prostate cancer tissues were used as positive controls to test hnRNPK antibody for IHC staining (Fig. S1A). Negative controls were created by replacing the primary antibody with non‐immune immunoglobulin G (IgG; DAKO, Glostrup, Copenhagen, Denmark) (Fig. S1B).

Heterogeneous nuclear ribonucleoprotein K expression in the bladder cancer specimens was blind‐quantified by two pathologists using a previously described scoring system 23. Briefly, the immunostaining intensity of each sample was graded as negative = 0, weak = 1, moderate = 2, or strong = 3 (Fig. S2). The proportion of positively staining cells was assessed as the percentage. The score was then calculated as the intensity score multiplied by the percentage of cells stained (score = intensity × % of positive cells). The samples were classed as low (score <140) or high (score ≥140) hnRNPK expression. Images were visualized using a Nikon ECLIPSE Ti (Fukasawa, Japan) microscope system and processed with Nikon software.

### Cell culture

The human bladder cancer cell lines UM‐UC‐3 and T24 (ATCC, Manassas, VA, USA) were used in this study. UM‐UC‐3 cells were cultured in DMEM (Gibco, Shanghai, China), whereas T24 cells were cultured in RPMI 1640 (Gibco). All medium was supplemented with 10% foetal bovine serum (Shanghai ExCell Biology, Shanghai, China) and 1% penicillin/streptomycin. The cells were grown in a humidified atmosphere of 5% CO_2_ at 37°C.

### RNA interference

Small interfering RNA (siRNA) oligos targeting hnRNPK (si‐hnRNPK; siRNA‐1: GGGUUGUAGAGUGCAUAAATT, siRNA‐2: GCCUCCAUCUAGAAGAGAUTT) or negative control siRNA were purchased from GenePharma (Shanghai, China). SiRNA transfections were performed with 75 nM siRNA and Lipofectamine RNAiMAX (Life Technologies, Thermo Fisher Scientific Inc., Waltham, Massachusetts, USA) as previously described 25. Mock cells were treated with RNAiMAX and cultured in Opti‐MEM for 6 hrs, but without siRNA.

### Stable hnRNPK knockdown cell lines

The pLKO.1 TRC cloning vector (Addgene plasmid: 10878) was used to generate short hairpin RNA (shRNA) against hnRNPK (ATGCCTCCATCTAGAAGAGAT) or the negative control (CCTAAGGTTAAGTCGCCCTCG). The lentivirus production and infection was conducted according to the manufacturer's protocol.

### Cell proliferation assay

The methyl thiazolyl tetrazolium (MTT; MTS, Promega, Shanghai, China) colorimetric assay was used to screen for cell viability. Mock cells and cells transfected with control or hnRNPK siRNA were seeded in 96‐well plates at 2 × 10^3^ cells per well. Then, the absorbance was measured at 490 nm over 5 days using a SpectraMax M5 unit (Molecular Devices).

For the colony formation assay, the cells were seeded in a 6‐well plate at a density of 1000 cells per well after siRNA transfection. Approximately 10 days later, the clones were washed with 1× PBS and stained with crystal violet for approximately 20 min. Finally, the clones were imaged and quantified.

For the cell cycle analysis, cells were harvested 48 hrs after transfection and fixed in 70% ice‐cold ethanol, followed by RNase A treatment, and stained with 50 μg/ml propidium iodide (PI) for DNA content analysis in a FACSCalibur BD flow cytometer (Franklin Lakes, New Jersey, USA). The data were collected and processed using BD FACSuite analysis software (Franklin Lakes, New Jersey, USA).

The ethynyl deoxyuridine (EdU) assay was performed according to the manufacturer's instructions (RiboBio, Guangzhou, China). At 24 hrs after transfection, cells were seeded at 1.5 × 10^4^ cells per well in a 48‐well plate. At 48 hrs after transfection, 50 μM EdU was added to the plate, incubated for 2 hrs with the cells, and the nuclei were stained with 4′,6‐diamidino‐2‐phenylindole. The images were captured using an Olympus laser scanning microscope system (Tokyo, Japan).

### Chemosensitivity assay

Mock and transfected cells were treated with 0, 0.5, 1, 1.5, 2 and 2.5 μg/ml cisplatin (Sigma‐Aldrich, St. Louis, Missouri, USA) for 48 hrs. Chemosensitivity was measured using the same method as the MTS assay. To calculate the median inhibitory concentration (IC_50_), data were fitted in GraphPad Prism 5 (GraphPad, San Diego, CA, USA) and a dose–response curve was plotted using the four‐parameter dose–response curve as follows: Y = bottom + (top − bottom)/(1 + 10^[(Log IC50‐X) × HillSlope)]^ 23.

### Apoptosis analysis

At 24 hrs after transfection with control or hnRNPK siRNA, mock cells and experimental cells were treated with 0 μg/ml or IC_50_ cisplatin for 24 hrs. The IC_50_ of cisplatin in the UM‐UC‐3 and T24 cells was 1.8 μg/ml and 1.3 μg/ml respectively. The cells were collected, washed with PBS, and apoptosis was analysed with annexin V–fluorescein isothiocyanate and PI staining (Biotool, Shanghai, China) in a FACSCalibur BD flow cytometer.

### Detection of caspase‐3/7 activity

Caspase‐3/7 activity was measured using a Caspase‐Glo 3/7 Assay kit (Promega) as previously described 23.

### RNA isolation and quantitative RT‐PCR

Total cellular RNA was extracted using TRIzol (Invitrogen, Waltham, Massachusetts, USA) according to the manufacturer's protocol and was used for reverse transcription with a PrimeScript RT‐PCR kit (TaKaRa Biotechnology, Dalian, China). RT‐qPCR was conducted using a standard SYBR Green PCR kit (Roche Penzberg, Upper Bavaria, Germany) protocol with a LightCycler 96 Real‐Time System (Roche). The relative expression was calculated using the comparative cycle threshold (2^−∆∆Ct^) method. The transcription level of GAPDH was used as the internal control. Table S2 lists the specific primers used.

### Western blotting

Western blotting was performed as previously described 23, 26. Primary antibodies specific to hnRNPK, XIAP‐associated factor 1 (XAF1), ERCC excision repair 4, endonuclease catalytic subunit (ERCC4), ERCC1, G0/G1 switch 2 (G0S2) (1:200; Santa Cruz Biotechnology), cyclin A2, cyclin D1, cyclin E2, cleaved caspase‐7, and GAPDH (1:1000; CST, Danvers, Massachusetts, USA) were used. The blots were then incubated with goat anti‐rabbit or anti‐mouse secondary antibody (CST) and visualized using enhanced chemiluminescence.

### Tumorigenesis study

The Sun Yat‐sen University Institutional Animal Care and Use Committee approved all of the animal care and experimental procedures. Male BALB/c nude mice (4–5 weeks old) were purchased from the Sun Yat‐sen University Experimental Animal Center and housed in specific pathogen–free barrier facilities. Cells (3 × 10^6^) were injected subcutaneously in to the right or left side of the dorsum; six mice were used. Tumour sizes were measured every 3 days. At 21 days post‐implantation, the mice were killed and the tumours were surgically dissected; tumour specimens were fixed in 4% paraformaldehyde.

### RNA sequencing analysis

Cells were transfected with si‐hnRNPK (mixture of siRNA‐1 and ‐2) or control siRNA for 48 hrs. Then, total RNA was extracted from cells using TRIzol (Invitrogen). Library construction and sequencing were performed by Annoroad Gene Technology (Beijing, China). The libraries were sequenced on an Illumina HiSeq 2500 platform and 100‐bp paired‐end reads were generated. All primary data in RNA sequencing (RNA‐seq) analysis have been uploaded to the Gene Expression Omnibus (GSE79832). Gene ontology (GO) pathway analysis was performed with Molecule Annotation System 3.0 (MAS 3.0; CapitalBio, Beijing, China).

### Chromatin immunoprecipitation

Chromatin immunoprecipitation (ChIP) was conducted using a EZ‐Magna ChIP A/G kit (Millipore, Billerica, Massachusetts, USA) according to the manufacturer's instructions. Cells were transfected with si‐hnRNPK (a mixture of siRNA‐1 and ‐2) or control siRNA for 48 hrs; 1 × 10^6^ cells were used for each reaction. The cells were fixed in 1% formaldehyde at room temperature for 10 min., and the nuclei were isolated with nuclear lysis buffer (Millipore) supplemented with protease inhibitor cocktail (Millipore). Chromatin DNA was sonicated and sheared to lengths between 200 and 1000 bp. The sheared chromatin was immunoprecipitated at 4°C overnight with anti‐hnRNPK antibody (ab39975; Abcam, Cambridge, Massachusetts, USA). Normal rabbit IgG and anti‐RNA polymerase II antibody (Millipore) were used as the negative and positive control respectively. Table S3 lists the ChIP‐qPCR primers. Heterogeneous nuclear ribonucleoprotein K and RNA polymerase II protein levels in the ChIP assays were detected by western blotting (Fig. S7).

### Statistical analyses

Data are presented as the mean ± S.D. of three independent experiments. Two‐tailed Student's *t*‐tests and one‐way anova were used to evaluate the data. Cumulative survival time was calculated using the Kaplan–Meier method and analysed by the log‐rank test. A multivariate Cox proportional hazards model was used to estimate the adjusted hazard ratios and 95% confidence intervals and to identify independent prognostic factors. All statistical analyses were performed with SPSS 19.0 (SPSS Inc., Chicago, IL, USA). Differences were considered statistically significant at *P* < 0.05 and *P* < 0.01.

## Results

### hnRNPK expression is increased in bladder cancer and associated with bladder cancer clinical characteristics

**Figure 1:**
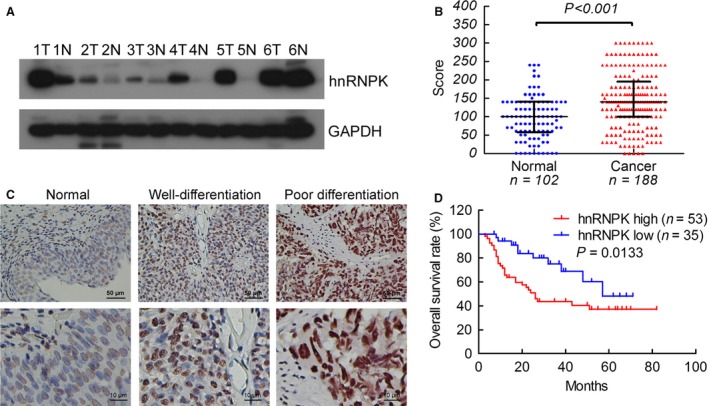
hnRNPK is up‐regulated in bladder cancer tissues. (**A**) Western blot detection of hnRNPK expression in six cases of bladder cancer tissue (T) and normal urothelium (N). (**B**) IHC expression of hnRNPK quantified by expression score (0–300) in normal urothelium and bladder cancer. (**C**) Representative IHC analysis of hnRNPK protein in normal, well‐differentiated, and poorly differentiated bladder cancer tissues. Magnification: ×400 (top) and ×1000 (bottom). (**D**) The overall survival rates of the 88 patients with bladder cancer were compared according to low‐ and high‐hnRNPK status. Statistical significance was determined using the log‐rank test. The samples were classed as low (score <140) or high (score ≥140) hnRNPK expression.

To detect hnRNPK expression in bladder cancer, we first performed western blot analysis on six cases of primary bladder cancer. Heterogeneous nuclear ribonucleoprotein K expression was up‐regulated in five of the cases as compared to the adjacent normal tissues (Fig. 1A). To further evaluate hnRNPK expression and its relationship with the clinical features of bladder cancer, we examined hnRNPK expression in 188 bladder cancer tissues and 102 normal tissues by IHC. Figure S1 shows the positive and negative controls for hnRNPK in IHC. Heterogeneous nuclear ribonucleoprotein K was mainly expressed in the nuclei of the bladder cancer cells and was significantly overexpressed in bladder cancer tissues as compared with normal tissues (score: 143.3 ± 5.7 *versus* 95.3 ± 5.8, *P* < 0.001, Fig. 1B, Fig. S3). Moreover, hnRNPK expression was obviously higher in poorly differentiated tissues as compared to well‐differentiated tissues (Fig. 1C). Clinicopathological correlation analysis revealed positive correlation between elevated hnRNPK levels with poor differentiation and advanced tumour stage (Table 1). There was no correlation between hnRNPK expression and tumour size or lymph node status.

**Table 1:** Relationship between hnRNPK expression and clinicopathological features of bladder caner

Characteristics	Cases (%)	Score	*P*‐value
Patients (*N*)	188		
Gender, *N* (%)			
Male	130 (69.1)	137.4 ± 6.7	0.1241
Female	58 (30.9)	156.6 ± 10.9	
Age (year)			
≤65	94 (50.0)	129.8 ± 8.2	0.0184^a^
>65	94 (50.0)	156.8 ± 7.8	
Pathologic tumour grade, *N* (%)			
Low grade	59 (29.8)	83.7 ± 7.2	<0.0001^a^
High grade	129 (70.2)	170.6 ± 6.4	
Tumour stage			
CIS,Ta,T1	56 (29.8)	123.0 ± 8.2	0.0209^a^
T2‐4	132 (70.2)	151.9 ± 7.3	
Patients (*N*)	116		
Tumour size *N* (%)			
≤3 cm	45 (38.8)	148.0 ± 9.7	0.4900
>3 cm	71 (61.2)	157.7 ± 9.3	
Lymphnodes status, *N* (%)			
Negative	99 (85.3)	153.8 ± 7.3	0.9621
Positive	17 (14.7)	154.7 ± 18.4	

a *P* < 0.05 is considered significant. The score is presented as the means ± SD of values obtained in three independent experiments.

### hnRNPK expression predicts disease prognosis

Kaplan–Meier survival analysis showed significantly reduced overall survival (*P* = 0.0133, median survival, 26 months) in patients with bladder cancer with increased hnRNPK expression as compared with the median overall survival of 57 months in patients with low hnRNPK immunostaining (Fig. 1D). To further evaluate the prognostic factors associated with overall survival in bladder cancer, we first carried out univariate analysis using age, sex, tumour stage, histological grade, node stage, tumour size and hnRNPK expression as parameters. Heterogeneous nuclear ribonucleoprotein K expression and nodal metastasis were significantly associated with overall survival (*P* = 0.017 and 0.020, respectively, Table 2). Moreover, the variables associated with survival by univariate analyses were adopted as covariates in the multivariate analyses, which revealed that high‐hnRNPK expression in addition to positive node stage was an independent predictor of overall survival (*P* = 0.013 and 0.013, respectively, Table S2). These findings clearly demonstrate the potential of hnRNPK as a marker of poor prognosis in bladder cancer.

**Table 2:** Univariate and multivariate analysis of factors associated with overall survival in bladder cancer

Variable	Univariate			Multivariate		
	HR^2^	95% CI	*P*	HR^2^	95% CI	*P*
Age, years (>65/≤65)	1.107	0.592–2.070	0.751			NA
Gender (female/male)	1.522	0.673–3.444	0.313			NA
Histological grade (high/low)	0.984	0.447–2.166	0.968			NA
Tumour stage (T2–T4/Ta–T1)	1.296	0.646–2.600	0.466			NA
Nodal metastasis (N1–N2/N0)	2.435	1.151–5.151	**0.020**	2.588	1.225–5.469	**0.013**
Tumour size (>3 cm/≤3 cm)	0.642	0.343–1.200	0.165			NA
hnRNPK (high/low)	2.391	1.167–4.899	**0.017**	2.487	1.212–5.103	**0.013**

Univariate and multivariate analysis. Cox proportional hazards regression model. Variables associated with survival by univariate analyses were adopted as covariates in multivariate analyses. Significant *P*‐values are shown in bold font. HR >1, risk for death increased; HR <1, risk for death reduced.

### hnRNPK knockdown inhibits bladder cancer cell proliferation by regulating the cell cycle

**Figure 2:**
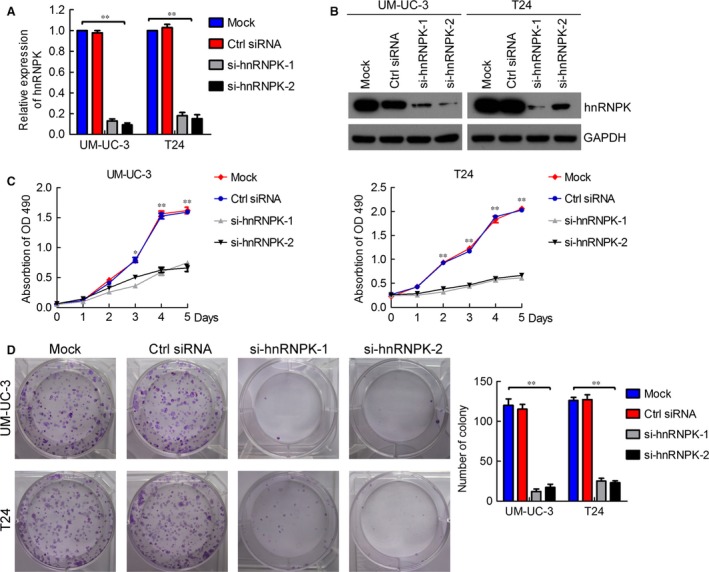
hnRNPK knockdown inhibits bladder cancer cell proliferation. (**A** and **B**) RT‐qPCR and western blotting verification of si‐hnRNPK knockdown efficiency in UM‐UC‐3 and T24 cells. (**C**) MTT assay evaluation of influence of hnRNPK knockdown on UM‐UC‐3 and T24 cell viability. (**D**) Colony formation assay determining the effect of hnRNPK knockdown in UM‐UC‐3 and T24 cells. The results are presented as the means ± S.D. of three independent experiments. *P < 0.05, **P < 0.01.

To study the role of hnRNPK in bladder cancer, we suppressed hnRNPK in bladder cancer cells *via* siRNA transfection. As shown in Figure 2A and B, hnRNPK was remarkably down‐regulated in UM‐UC‐3 and T24 cells transfected with the hnRNPK siRNAs as compared with those transfected with control siRNA at both mRNA and protein level, as confirmed by RT‐qPCR and western blotting. Moreover, hnRNPK knockdown using the two si‐hnRNPKs significantly inhibited tumour cell growth as compared to the mock and control cells (Fig. 2C). Consistent with our cell growth data, hnRNPK knockdown cells formed significantly fewer colonies than the mock and control cells (Fig. 2D). Furthermore, hnRNPK overexpression in UM‐UC‐3 cells by transfection revealed that hnRNPK upregulation promoted tumour cell growth and colony formation (Fig. S4A–E).

**Figure 3:**
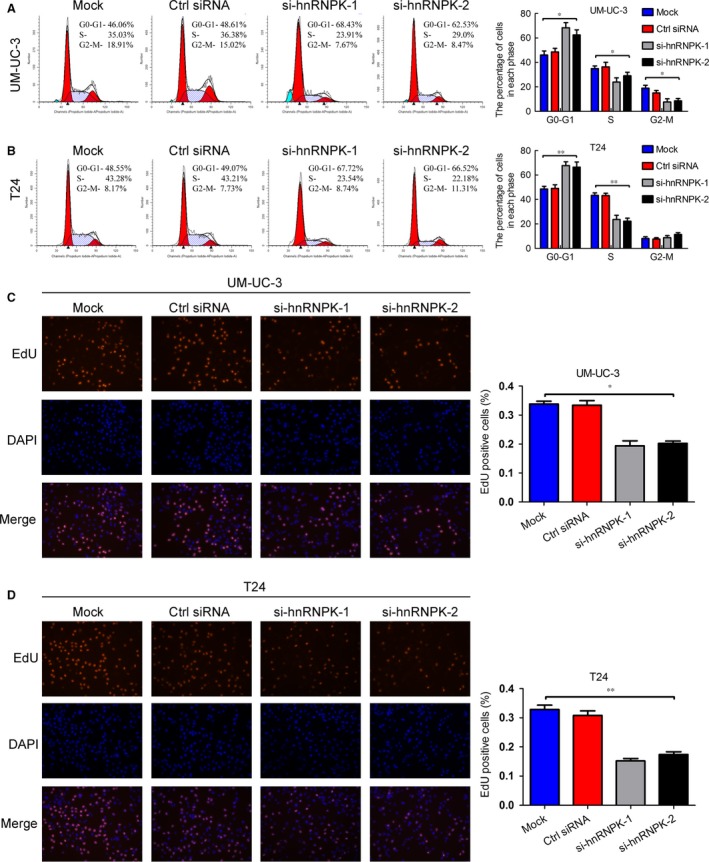
hnRNPK knockdown induces G0/G1 arrest in bladder cancer cells. (**A** and **B**) Flow cytometry analysis of UM‐UC‐3 and T24 cells transfected with si‐hnRNPK or control siRNA for 48 hrs. The percentages (%) of cell populations at different stages of the cell cycle are listed in the panels. All histograms show the percentage (%) of cell populations from three independent experiments. (**C** and **D**) EdU assay measurement of the cell population in the S phase. Blue, nucleus; red, S‐phase cells (EdU‐positive). Histological analysis of the percentage of EdU‐positive cells in control and hnRNPK knockdown cells is shown. The results are presented as the means ± S.D. of three independent experiments. *P < 0.05, **P < 0.01.

Next, we performed flow cytometry and EdU assays to characterize whether hnRNPK was involved in the cell cycle. Interestingly, hnRNPK silencing dramatically increased the cell population in the G0/G1 phase, whereas it reduced the cell population in the S and G2/M phases (Fig. 3A and B). On the contrary, hnRNPK up‐regulation decreased the cell population in the G0/G1 phase and increased the cell population in the S and G2/M phases (Fig. S4F–G). The EdU assay showed that hnRNPK knockdown significantly decreased the cell population in the S phase (Fig. 3C and D). Collectively, these results indicate that hnRNPK knockdown inhibits bladder cancer cell proliferation by inducing G0/G1 arrest.

### hnRNPK regulates apoptosis and chemoresistance in bladder cancer cells

**Figure 4:**
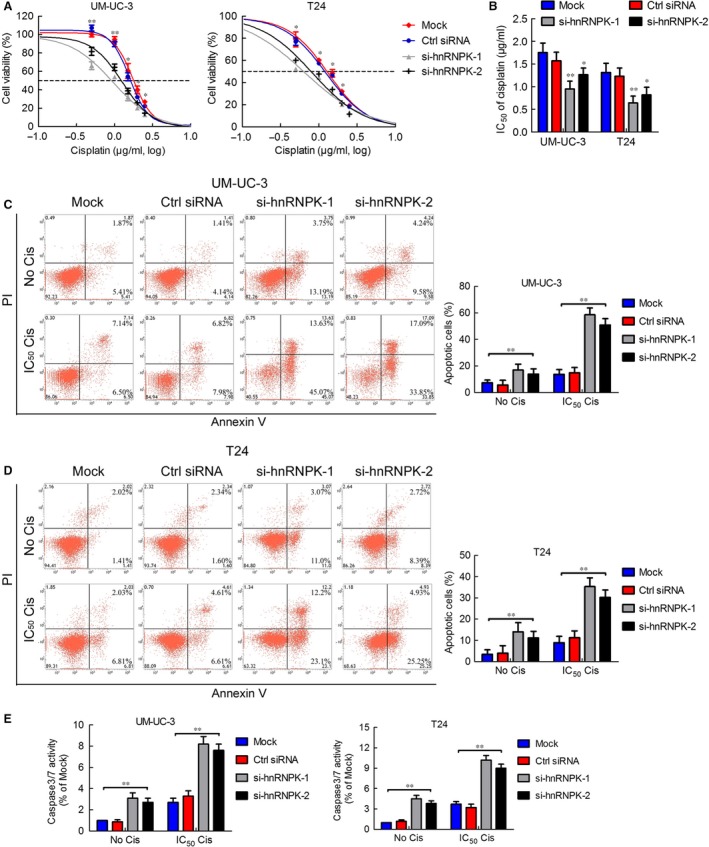
hnRNPK regulates apoptosis and chemoresistance in bladder cancer cells. (**A**) MTT assay analysis of viability of cells transfected with si‐hnRNPK or control siRNA and treated with cisplatin for 48 hrs. (**B**) The four‐parameter logistic curve (best‐fit solution, non‐linear regression dynamic fitting) and normality tests were used to determine the IC_50_. (**C** and **D**) At 24 hrs after transfection with control siRNA or si‐hnRNPK, UM‐UC‐3 cells were treated with 0 or 1.8 μg/ml cisplatin for 24 hrs; T24 cells were treated with 0 or 1.3 μg/ml cisplatin. The percentage of apoptotic cells was analysed by flow cytometer. Histograms show the percentage (%) of late and early apoptotic cells from three independent experiments. (**E**) Caspase‐3/7 activity assay was performed on UM‐UC‐3 and T24 cells transfected with control sRNA or si‐hnRNPK and treated with or without the cisplatin IC_50_ of the parental cells for 24 hrs. Relative caspase‐3/7 activity is indicated as the percentage of untreated parental cells. The results are presented as the means ± S.D. of three independent experiments. *P < 0.05, **P < 0.01.

We investigated the role of hnRNPK in apoptosis and chemoresistance *via* the MTT assay and flow cytometry. As shown in Figure 4A and B, cells transfected with si‐hnRNPK exhibited lower resistance to cisplatin and the cisplatin IC_50_ than the mock and control cells. However, hnRNPK overexpression increased UM‐UC‐3 cell resistance to cisplatin and the cisplatin IC_50_ (Fig. S5A and B). We quantified apoptosis by staining cells with annexin V and PI. Heterogeneous nuclear ribonucleoprotein K knockdown induced apoptosis and significantly increased the percentage of apoptotic cells under cisplatin treatment (Fig. 4C and D). Heterogeneous nuclear ribonucleoprotein K up‐regulation decreased the percentage of apoptotic cells under cisplatin treatment in an obvious manner (Fig. S5C and D). Compared with the mock and control cells, caspase‐3/7 activity was up‐regulated in hnRNPK knockdown cells and was obviously increased when the cells were treated with cisplatin (Fig. 4E). These results suggest that hnRNPK plays a critical role in bladder cancer cell apoptosis and chemoresistance to cisplatin.

### hnRNPK down‐regulation suppresses bladder cancer cell tumorigenicity *in vivo*

**Figure 5:**
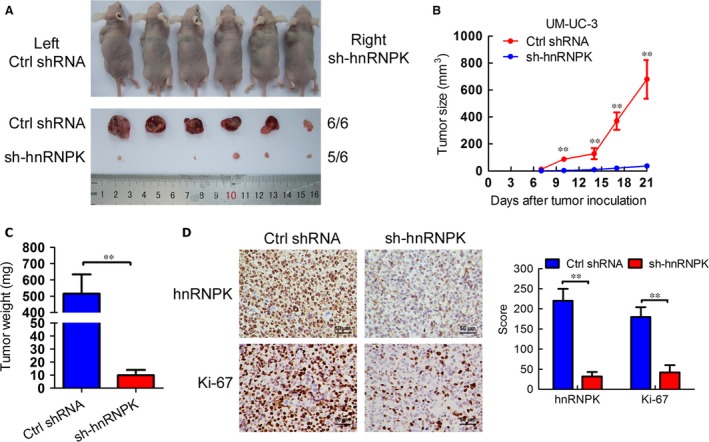
hnRNPK down‐regulation suppresses bladder cancer cell tumorigenicity *in vivo*. (**A**) Animals and tumours in this study. (**B**) The tumour growth volume was measured every 3 days. The results are presented as the means ± S.D. (*n* = 6). (**C**) Tumour weights were measured after the tumours were surgically dissected. (**D**) IHC examination of tumour hnRNPK and Ki67 expression. Histogram shows the IHC score in control and hnRNPK knockdown groups. **P < 0.01.

To further explore the effects of hnRNPK in bladder cancer tumorigenesis *in vivo*, we stably suppressed hnRNPK in UM‐UC‐3 cells by lentiviral transfection (Fig. S6). Next, the hnRNPK stable knockdown or sh‐control cells were subcutaneously injected into BALB/c nude mice and the tumour growth activity was measured. Interestingly, the growth of tumours derived from the hnRNPK knockdown group was prominently suppressed as compared with the control group at 10 days after inoculation (Fig. 5A and B). The size and weight of tumours from the hnRNPK knockdown group were significantly lower than that of the control group (Fig. 4B and C). Moreover, the tumours derived from the hnRNPK knockdown group had lower expression of hnRNPK and the proliferation marker Ki67 than the control group (Fig. 5D and E). These results indicate that hnRNPK promotes bladder cancer cell tumorigenicity *in vivo*.

### The target genes of hnRNPK are identified in bladder cancer

**Figure 6:**
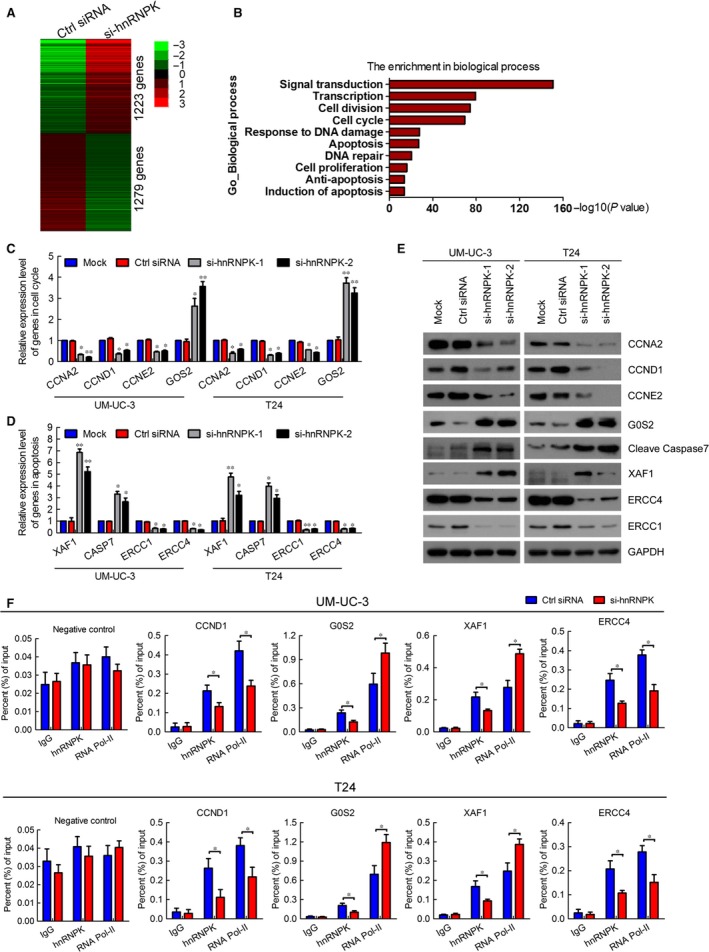
Identification of target genes of hnRNPK in bladder cancer. (**A**) Heat map representing unsupervised hierarchical clustering of mRNA expression levels in UM‐UC‐3 cells transfected with control siRNA or si‐hnRNPK for 48 hrs. Each column represents the indicated sample; each row indicates one mRNA. Red and green indicate high and low expression respectively. (**B**) GO pathway analysis was used to identify the enrichment of biological processes. (**C** and **D**) RT‐qPCR verification of differentially expressed genes in the RNA‐seq of UM‐UC‐3 and T24 cells. The results are presented as the means ± S.D. of three independent experiments. (**E**) Western blot detection of the expression of hnRNPK target genes. GAPDH was used as the internal control. (**F**) ChIP analysis of IgG, hnRNPK, and RNA polymerase II status of candidate hnRNPK target genes in UM‐UC‐3 cells after knockdown assay. The values are normalized to input and presented as the mean ± S.D. *P < 0.05, **P < 0.01.

Heterogeneous nuclear ribonucleoprotein K is mainly expressed in the nuclei of bladder cancer cells. To investigate the mechanism of hnRNPK in bladder cancer, we performed RNA‐seq to analyse the changes in target gene mRNA levels between UM‐UC‐3 cells that had been transfected with si‐hnRNPK or control siRNA. The hnRNPK knockdown group had 1223 up‐regulated genes and 1279 down‐regulated genes compared with the control group (Fig. 6A). Gene ontology pathway analysis revealed that the genes regulated by hnRNPK were enriched in signal transduction, transcription, cell cycle, response to DNA damage and apoptosis (Fig. 6B). Next, we validated the expression of these genes in UM‐UC‐3 and T24 cells transfected with control siRNA or si‐hnRNPK by RT‐qPCR. Compared with the mock and control cells, the mRNA expression of cyclin A2, cyclin D1, and cyclin E2, which promote the cell cycle, were significantly decreased in hnRNPK‐silenced cells, whereas the mRNA expression of G0S2, which arrests the cell cycle, was obviously increased in hnRNPK‐silenced cells (Fig. 6C). Moreover, hnRNPK knockdown inhibited the genes of chemoresistance, such as *ERCC1* and *ERCC4*. However, hnRNPK knockdown promoted the genes of apoptosis, such as that for caspase‐7 (*CASP7*), and *XAF1* (Fig. 6D). The protein expression of these genes was consistent with the change in mRNA level (Fig. 6E). ChIP‐qPCR performed to confirm whether hnRNPK directly regulates these genes determined that hnRNPK knockdown decreased the levels of hnRNPK in the promoter regions of cyclin D1, *G0S2*,* XAF1* and *ERCC4*, but not in the negative control or in other genes. Moreover, RNA polymerase II levels were decreased in the promoter regions of cyclin D1 and *ERCC4*, but were increased in the promoter regions of *G0S2* and *XAF1* (Fig. 6F, Figs S8 and S9). Taken together, these data indicate that hnRNPK regulates target genes in bladder cancer by directly mediating transcription.

## Discussion

The hnRNP family members play key roles in several biological functions, such as chromosome remodelling, cellular signal transduction, and transcriptional and translational regulation. Emerging evidence shows that aberrant up‐regulation of hnRNP is involved in tumorigenesis. hnRNPA/B proteins are overexpressed in hepatocellular carcinoma and non‐small cell lung cancer, and indicate poor prognosis 27, 28. Balasubramani *et al*. showed that hnRNPF was a potential marker for colorectal cancer progression 29. Aberrant hnRNPK expression in the cytoplasm has been reported in pancreatic cancer 15, colorectal cancer 16, prostate cancer 17, and renal cell carcinoma 18, and was associated with poor clinical prognosis. However, the expression and biological functions of hnRNPK underlying tumorigenesis and progression in bladder cancer remain unknown. In this study, we found that hnRNPK is mainly expressed in the nucleus and rarely detected in the cytoplasm of bladder cancer cells. We show for the first time that increased expression of nuclear hnRNPK in bladder cancer cells is positively correlated with poor differentiation and advanced tumour stage. Furthermore, high nuclear hnRNPK expression was associated with poor prognosis and served as an independent predictor of overall survival in bladder cancer. Consistent with our findings, Barboro *et al*. found that high‐hnRNPK expression in prostate cancer was closely associated with Gleason score and poor prognosis 17. Taken together, high‐hnRNPK expression levels may serve as a novel prognostic marker for bladder cancer.

As reported previously, hnRNPK has been implicated in several biological functions crucial for cancer development 30, including proliferation 15, 31, 32, metastases 19, 20, angiogenesis 33 and neuroendocrine differentiation 34. Here, we discovered that hnRNPK knockdown significantly inhibited bladder cancer cell proliferation *in vitro* and tumour growth *in vivo* by inducing G0/G1 arrest. Supporting our findings, recent studies have found that hnRNPK down‐regulation suppressed cell proliferation in pancreatic cancer 15 and renal cell carcinoma 18 *in vitro*, but the underlying mechanism remains largely unknown. Through RNA‐seq analysis and ChIP, we determined that hnRNPK regulates cyclin D1 and G0S2 transcription. Cyclin D1, a key regulator in G1‐to‐S‐phase transition, is overexpressed in bladder cancer and associated with poor prognosis 35, 36, 37. Several studies have revealed that G0S2 suppresses oncogenic transformation and induces apoptosis in cancer cells 38, 39. These data strongly suggest that hnRNPK regulates the cell cycle of bladder cancer cells mainly by transcriptional regulation of cyclin D1 and G0S2.

In this study, we found that hnRNPK knockdown increased apoptosis and sensitized bladder cancer cells to cisplatin. Mechanistically, we first demonstrated that hnRNPK maintained anti‐apoptosis and promoted chemoresistance in bladder cancer cells *via* transcriptional regulation of *XAF1* and *ERCC4*. A recent study revealed that *XAF1* is down‐regulated in bladder cancer and associated with good prognosis 40, 41. Zhu *et al*. 42 found that *XAF1* induces apoptosis, inhibits angiogenesis, and inhibits tumour growth in hepatocellular carcinoma. Similarly, hnRNPK suppresses apoptosis independent of p53 status in hepatocellular carcinoma by increasing *XIAP* transcription 22. However, hnRNPK knockdown did not affect *XIAP* mRNA levels in bladder cancer cells (data not shown), suggesting that the mechanism of hnRNPK on apoptosis differs between cancers. ERCC4 plays an essential role in the nucleotide excision repair pathway and is involved in chemoresistance in several cancers 43, 44, 45, including bladder cancer 46, 47. Consistent with our findings, hnRNPK down‐regulation by a mitogen‐activated extracellular signal‐regulated kinase kinase inhibitor increased the radiotherapy sensitivity in malignant melanoma cells 48. Collectively, these findings indicate that hnRNPK enhances bladder cancer cell anti‐apoptosis and chemoresistance to cisplatin by regulating *XAF1* and *ERCC4* and that it may be a potential target for drug development.

Heterogeneous nuclear ribonucleoprotein K is closely implicated in various molecular functions in cancer, such as transcription, mRNA stability, splicing, translation and protein interaction 30, 49, 50. Several studies have found that hnRNPK transcription activates several important oncogenes, including c‐*SRC* and c‐*MYC* 51, 52. As hnRNPK is mainly expressed in the nuclei of bladder cancer cells, we focused on its function in the nucleus and used RNA‐seq to explore the target genes. Interestingly, the genes regulated by hnRNPK were mainly enriched in signal transduction, cell cycle, response to DNA damage and apoptosis, which is consistent with cellular function in bladder cancer. As hnRNPK binds tightly to polyC‐DNA 30, we performed a ChIP assay and designed primers to detect such DNA fragments on the gene promoters. We found that hnRNPK regulated the transcription of cyclin D1 (*CCND1*), *G0S2*,* XAF1* and *ERCC4* by binding their promoters. These results suggest that hnRNPK plays an oncogenic role in bladder cancer by directly mediating these genes. However, the function of hnRNPK in mRNA splicing and the cytoplasm remains largely unknown, and further investigation is underway to elucidate these key questions in bladder cancer.

In conclusion, it is our novel discovery that hnRNPK is up‐regulated in bladder cancer and correlates with poor prognosis. Moreover, hnRNPK promotes bladder cancer cell proliferation, anti‐apoptosis and chemoresistance to cisplatin by regulating a series of genes at transcriptional level. Therefore, hnRNPK is a potential biomarker for bladder cancer and a promising target for drug development.

## Conflict of interest

The authors declare no conflict interest.

## Supporting information


**Figure S1** (A) Human prostate cancer tissues as the positive control to test IHC hnRNPK antibody staining.
**Figure S2** The immunostaining intensity of each sample was graded as negative = 0, weak = 1, moderate = 2, or strong = 3. Representative samples are shown at ×400 magnification.
**Figure S3** Immunocytochemical analyses of hnRNPK expression in UM‐UC‐3 and T24 cells.
**Figure S4** hnRNPK overexpression promotes bladder cancer cell proliferation by regulating the cell cycle.
**Figure S5** hnRNPK promotes anti‐apoptosis and chemoresistance to cisplatin in bladder cancer cells.
**Figure S6** Western blot verification of hnRNPK stable knockdown efficiency in UM‐UC‐3 cells by lentivirus.
**Figure S7** Western blot detection of hnRNPK and RNA polymerase II levels in the ChIP assays.
**Figure S8** ChIP analysis of IgG, hnRNPK, and RNA polymerase II status of candidate hnRNPK target genes in UM‐UC‐3 and T24 cells in DNA gel.
**Figure S9** ChIP analysis of IgG, hnRNPK, and RNA polymerase II status of candidate hnRNPK target genes in UM‐UC‐3 and T24 cells after knockdown assay.
**Table S1** Characteristics of patients and tumours in tissue specimens.
**Table S2** List of primer sequences for PCR studies.
