# Supplementary material for: Heterogeneous nuclear ribonucleoprotein K is associated with poor prognosis and regulates proliferation and apoptosis in bladder cancer
Source: J Cell Mol Med. 2016 Nov 10;21(7):1266–79. doi: 10.1111/jcmm.12999 (PMC5487918; doi:10.1111/jcmm.12999)
Supplement: Supplementary file 1 — Figure S1 (A) Human prostate cancer tissues as the positive control to test IHC hnRNPK antibody staining. Figure S2 The immunostaining intensity of each sample was graded as negative = 0, weak = 1, moderate = 2, or strong = 3. Representative samples are shown at ×400 magnification. Figure S3 Immunocytochemical analyses of hnRNPK expression in UM‐UC‐3 and T24 cells. Figure S4 hnRNPK overexpression promotes bladder cancer cell proliferation by regulating the cell cycle. Figure S5 hnRNPK promotes anti‐apoptosis and chemoresistance to cisplatin in bladder cancer cells. Figure S6 Western blot verification of hnRNPK stable knockdown efficiency in UM‐UC‐3 cells by lentivirus. Figure S7 Western blot detection of hnRNPK and RNA polymerase II levels in the ChIP assays. Figure S8 ChIP analysis of IgG, hnRNPK, and RNA polymerase II status of candidate hnRNPK target genes in UM‐UC‐3 and T24 cells in DNA gel. Figure S9 ChIP analysis of IgG, hnRNPK, and RNA polymerase II status of candidate hnRNPK target genes in UM‐UC‐3 and T24 cells after knockdown assay. Table S1 Characteristics of patients and tumours in tissue specimens. Table S2 List of primer sequences for PCR studies. [file JCMM-21-1266-s001.docx]

**Heterogeneous nuclear ribonucleoprotein K (hnRNPK) is associated with poor prognosis and regulates proliferation and apoptosis in bladder cancer**

Xu Chen^1, 2, #^, Peng Gu^1, 2, #^, Ruihui Xie^1, 2, #^, Jinli Han^1^, Hao Liu^1^, Bo Wang^1, 2^, Weibin Xie^1, 2^, Weijie Xie^1^, Guangzheng Zhong^1^, Changhao Chen^1^, Shujie Xie^1^, Ning Jiang^1^, Tianxin Lin^1,2,*^ & Jian Huang^1,*^

^1^ Department of Urology, Sun Yat-sen Memorial Hospital, Sun Yat-sen University, Guangzhou, China

^2^ Guangdong Provincial Key Laboratory of Malignant Tumor Epigenetics and Gene Regulation, Sun Yat-Sen Memorial Hospital, Sun Yat-Sen University,Guangzhou, China

^#^ These three authors (Xu Chen, Peng Gu, Ruihui Xie) contributed equally to this work.

Corresponding authors Address: Department of Urology, Sun Yat-sen Memorial Hospital, 107th Yanjiangxi Road, Guangzhou, China. Jian Huang, Tel: 86-13600054833, fax: 86-20-81332336. E-mail: [urolhj@sina.com](mailto:urolhj@sina.com) and Tianxin Lin, Tel: 86-13724008338, fax: 86-20-81332336. E-mail: [tianxinl@sina.com](mailto:tianxinl@sina.com).

**Supplementary information**

**Material and methods**

***Immunofluorescence analysis***

UM-UC-3 and T24 cells were cultured on glass slides overnight, fixed in 4% paraformaldehyde for 30 min, incubated with 0.5% Triton-X in PBS for 10 min, and blocked in 5% bovine serum albumin for 30 min. After incubation, the cells were stained with a primary antibody against hnRNPK (1:50; Santa Cruz Biotechnology) for 12 h at 4°C, then washed and incubated with Alexa Fluor–conjugated goat anti-mouse IgG (1:200; Santa Cruz Biotechnology) for 1 h at room temperature. After washing in PBS, the cells were coverslipped with a mounting medium containing 4',6-diamidino-2-phenylindole (DAPI). The stained cells were examined under a confocal laser scanning microscope (Zeiss).

***hnRNPK overexpression in UM-UC-3 cells***

The hnRNPK coding sequence was PCR-amplified and cloned in a pcDNA3.0 plasmid (Invitrogen, Life Technologies). Transfections were performed with 1.5 μg pcDNA3.0 or pcDNA3.0-hnRNPK and X-tremeGENE (Roche) in 6-well plates according to the manufacturer’s instructions. After 24 h, the transfected cells were subjected to the function assays as described in the main text.

**Figure legends**

**Supplementary Fig. S1** (**A**) Human prostate cancer tissues as the positive control to test IHC hnRNPK antibody staining. (**B**) Negative controls created by replacing the primary antibody with nonimmune IgG in normal urothelium tissue and bladder cancer tissue. Representative samples are shown at ×400 magnification.

**Supplementary Fig. S2** The immunostaining intensity of each sample was graded as negative = 0, weak = 1, moderate = 2, or strong = 3. Representative samples are shown at ×400 magnification.

**Supplementary Fig. S3** Immunocytochemical analyses of hnRNPK expression in UM-UC-3 and T24 cells. The nuclei were stained with DAPI (blue); hnRNPK was stained with Alexa Fluor (red).

**Supplementary Fig. S4** hnRNPK overexpression promotes bladder cancer cell proliferation by regulating the cell cycle. (**A**) RT-qPCR and (**B**) western blot verification of hnRNPK overexpression efficiency in UM-UC-3 cells transfected with hnRNPK or control pcDNA vector. (**C**) MTT assay measurement of influence of hnRNPK overexpression on UM-UC-3 cell viability. (**D and E**) Effect of hnRNPK overexpression on UM-UC-3 cell colony formation ability. (**F and G**) Flow cytometry analysis of UM-UC-3 cells transfected with hnRNPK or control pcDNA vector for 48 h. Percentages (%) of cell populations at different stages of the cell cycle are listed in the panels. Histogram shows the percentage (%) of cell populations from three independent experiments. The results are presented as the means ± S.D. **P* < 0.05, ***P* < 0.01.

**Supplementary Fig. S5** hnRNPK promotes anti-apoptosis and chemoresistance to cisplatin in bladder cancer cells. (**A**) MTT assay analysis of viability of cells transfected with hnRNPK or control pcDNA vector and treated with cisplatin for 48 h. (**B**) The four-parameter logistic curve (best-fit solution, nonlinear regression dynamic fitting) and normality tests were used to determine the IC_50_. (**C and D**) At 24 h after transfection, UM-UC-3 cells were treated with 0 or 1.8 μg/ml cisplatin for 24 h. The percentage of apoptotic cells was analyzed by flow cytometer. Histogram shows the percentage (%) of late and early apoptotic cells from three independent experiments. The results are presented as the means ± S.D. **P* < 0.05.

**Supplementary Fig. S6** Western blot verification of hnRNPK stable knockdown efficiency in UM-UC-3 cells by lentivirus.

**Supplementary Fig. S7** Western blot detection of hnRNPK and RNA polymerase II levels in the ChIP assays.

**Supplementary Fig. S8** ChIP analysis of IgG, hnRNPK, and RNA polymerase II status of candidate hnRNPK target genes in UM-UC-3 and T24 cells in DNA gel.

**Supplementary Fig. S9** ChIP analysis of IgG, hnRNPK, and RNA polymerase II status of candidate hnRNPK target genes in UM-UC-3 and T24 cells after knockdown assay. Values are normalized to input and presented as the means ± S.D. **P* < 0.05. CCNA2, cyclin A2; CCNE2, cyclin E2.

**Figures**

**Supplementary Figure S1**


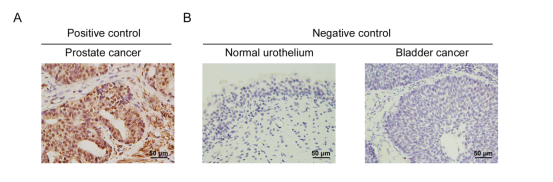


**Supplementary Figure S2**

**
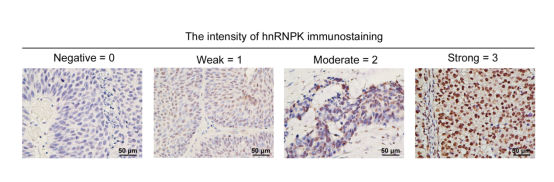
**

**Supplementary Figure S3**

**
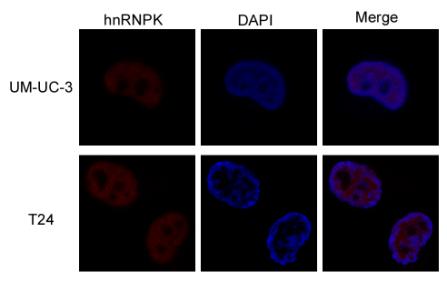
**

**Supplementary Figure S4**

**
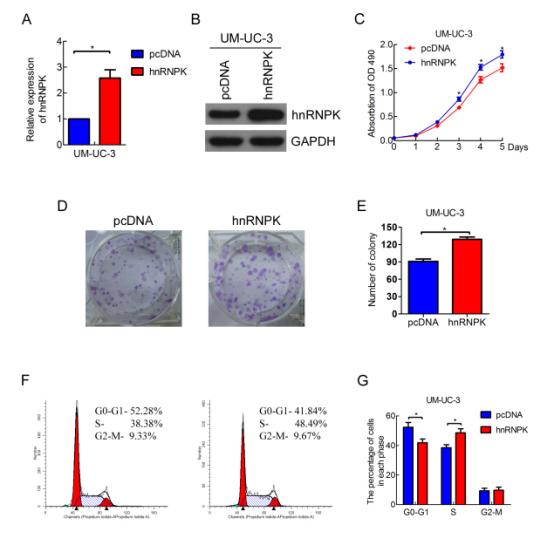
**

**Supplementary Figure S5**

**
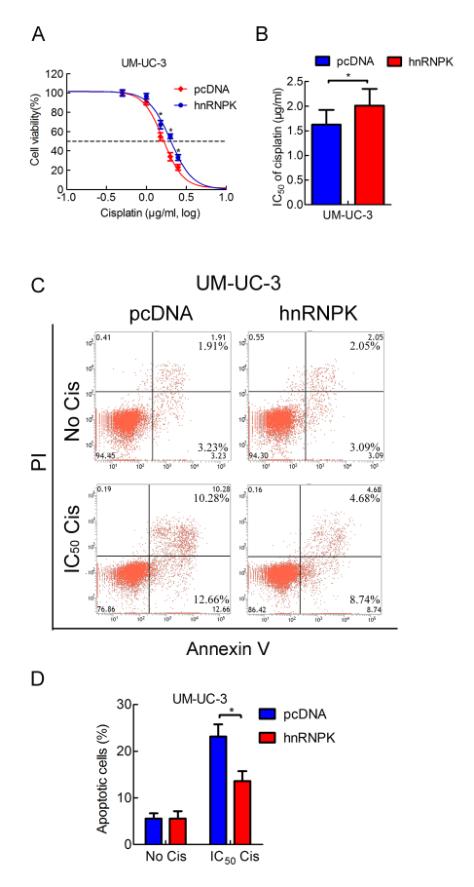
**

**Supplementary Figure S6**

**
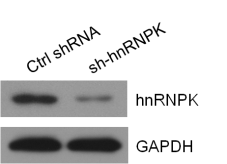
**

**Supplementary Figure S7**

**
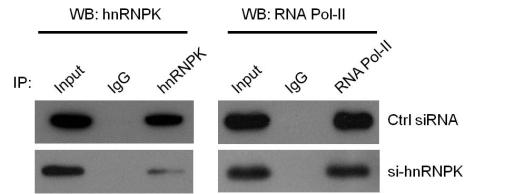
**

**Supplementary Figure S8**


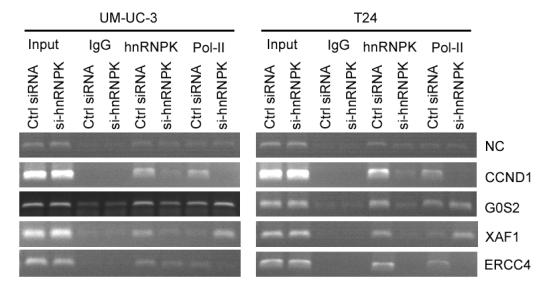


**Supplementary Figure S9**


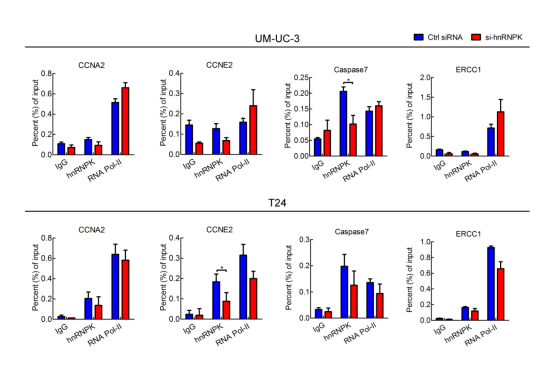


**Tables**

**Supplementary Table S1** Characteristics of patients and tumors in tissue specimens

|  | | Tumor tissues | | Tumor tissues for survival analysis |
| --- | --- | --- | --- | --- |
| **Patients(N)** | | 188 | | 88 |
| **Gender N(%)** | |  | |  |
| Male | | 130(69.1) | | 77(87.5) |
| Female | | 58(30.9) | | 11(12.5) |
| **Age(Year)** | |  | |  |
| Median(range) | | 65.5(37-85) | | 65.5(44-85) |
| Mean±SD | | 64.6±11.2 | | 66.2±10.7 |
| **Pathologic tumor grade** **N (%)** |  | |  |  |
| Low grade | | 59(29.8) | | 22(25.0) |
| High grade | | 129(70.2) | | 66(75.0) |
| **NMIBC** | | 56(29.8) | | 28(31.8) |
| pTa | | 5(2.7) | | 2(2.2) |
| pTis | | 5(2.7) | | 5(5.7) |
| pT1 | | 46(24.4) | | 21(23.9) |
| **MIBC** | | 132(70.2) | | 60(68.2) |
| pT2 | | 72(38.3) | | 24(27.3) |
| pT3 | | 48(25.5) | | 24(27.3) |
| pT4 | | 12(6.4) | | 12(13.6) |
| **Patients(N)** | | 116 | | 88 |
| **Tumor size N(%)** | |  | |  |
| ≤3cm | | 45(38.8) | | 39(44.3) |
| >3cm | | 71(61.2) | | 49(55.7) |
| **Lymphnodes status N(%)** | |  | |  |
| Negative | | 99(85.3) | | 76(86.4) |
| Positive | | 17(14.7) | | 12(13.6) |

**Supplementary Table S2** List of primer sequences for PCR studies.

| Target | Forward Sequence (5’-3’) | Reverse Sequence (5’-3’) |
| --- | --- | --- |
| **Primer for RT-qPCR**  hnRNPK | CCTATGACAGAAGAGGGAGAC | CCCTGTGGTTCATAAGCCATC |
| GAPDH  CCNA2 | CAAGGCTGAGAACGGGAAG  CCTCGTGGACTGGTTAGTTGA | TGAAGACGCCAGTGGACTC  AGGCTAACAGCATAGCAGCAG |
| CCND1 | CAAATGTGTGCAGAAGGAGGT | GAAGCGGTCCAGGTAGTTCA |
| CCNE2 | CGTTTACAAGCTAAGCAGCAG | CCTGGGTAGTTTTCCTCTTC |
| G0S2 | AAGGAGATGATGGCCCAGAAG | GAAGGGGCTGCACACAGTCTC |
| CASP7 | GCACGGAAAAGACCTGGAAATC | CACAGGGGATCTGCTTCTTCT |
| XAF1 | CCTAGAGGAGATAAAGCAGCC | GAAGCTAACCACCGGCATTTC |
| ERCC1 | AGATGTGTATCCTGGCCGACT | GGGAGACGAAGTCCTGCTCTA |
| ERCC4 | CCGTGCTTCTGATTGAGTTTG | CCGTAGTCTGGGGAAGTGAAG |
| **Primer for cloning the coding sequence of hnRNPK**  **Primer for ChIP-qPCR**  negative control  CCNA2  CCND1  CCNE2  G0S2  CASP7  XAF1  ERCC1  ERCC4 | ATGGAAACTGAACAGCCAGAAG  GTAATCAGGAAACTGCATAC  CACTTAAGCTAACTAGACGTCCC  gcagtgtcaggagacattcttg  gctcaggaaagacctgggttc  AAGGTGACAACCCTTCCGAAT  GAATCCGTGTGTGTGGTGGGT  AATTCTCTGGGCCTGCTTGTC  AATCAGAGAGCTGCAAGTTAG  GCTACTAACTCTTGGCGCATT | TTAGAATCCTTCAACATCTGCAT  CTCAAGACTCAATAGTGATC  TTTAAGCTGAGCCACCTAGTGA  ctgtgggaagttcagcagcat  cataacagggggacacgactc  GCGGCAACCTTCTTACTGGTG  CGCATTCCAGTTTACCCTGAC  CAATCTGGTGTGTATCCTCCT  GGAGGAAGGGACATCTGAGCA  TAGCCTCAAGCTGCCAAAAAG |
